# A multimedia consent tool for research participants in the Gambia: a randomized controlled trial

**DOI:** 10.2471/BLT.14.146159

**Published:** 2015-03-23

**Authors:** Muhammed Olanrewaju Afolabi, Nuala McGrath, Umberto D’Alessandro, Beate Kampmann, Egeruan B Imoukhuede, Raffaella M Ravinetto, Neal Alexander, Heidi J Larson, Daniel Chandramohan, Kalifa Bojang

**Affiliations:** a Medical Research Council Unit, Atlantic Road, Fajara, PO Box 273, Banjul, the Gambia.; b University of Southampton, Southampton, England.; c The Jenner Institute, University of Oxford, Oxford, England.; d Institute of Tropical Medicine, Antwerp, Belgium.; e London School of Hygiene & Tropical Medicine, London, England.

## Abstract

**Objective:**

To assess the effectiveness of a multimedia informed consent tool for adults participating in a clinical trial in the Gambia.

**Methods:**

Adults eligible for inclusion in a malaria treatment trial (*n* = 311) were randomized to receive information needed for informed consent using either a multimedia tool (intervention arm) or a standard procedure (control arm). A computerized, audio questionnaire was used to assess participants’ comprehension of informed consent. This was done immediately after consent had been obtained (at day 0) and at subsequent follow-up visits (days 7, 14, 21 and 28). The acceptability and ease of use of the multimedia tool were assessed in focus groups.

**Findings:**

On day 0, the median comprehension score in the intervention arm was 64% compared with 40% in the control arm (*P* = 0.042). The difference remained significant at all follow-up visits. Poorer comprehension was independently associated with female sex (odds ratio, OR: 0.29; 95% confidence interval, CI: 0.12–0.70) and residing in Jahaly rather than Basse province (OR: 0.33; 95% CI: 0.13–0.82). There was no significant independent association with educational level. The risk that a participant’s comprehension score would drop to half of the initial value was lower in the intervention arm (hazard ratio 0.22, 95% CI: 0.16–0.31). Overall, 70% (42/60) of focus group participants from the intervention arm found the multimedia tool clear and easy to understand.

**Conclusion:**

A multimedia informed consent tool significantly improved comprehension and retention of consent information by research participants with low levels of literacy.

## Résumé

**Objectif:**

Évaluer l'efficacité d'un outil multimédia de consentement éclairé pour les adultes participant à un essai clinique en Gambie.

**Méthodes:**

Les adultes éligibles à la participation à un essai de traitement du paludisme (*n* = 311) ont été randomisés pour le recueil des informations nécessaires au consentement éclairé, au moyen d'un outil multimédia (branche d'intervention) ou bien de la procédure standard (branche témoin). Un questionnaire audio informatisé a été utilisé pour évaluer la compréhension du consentement éclairé par les participants, immédiatement après l'obtention de ce consentement (jour 0) ainsi que lors des visites de suivi ultérieures (jours 7, 14, 21 et 28). L'acceptabilité et la facilité d'utilisation de cet outil multimédia ont été évaluées dans des groupes de discussions.

**Résultats:**

Au jour 0, le score médian de compréhension dans la branche d'intervention était de 64% contre 40% dans la branche témoin (*P* = 0,042). La différence est restée significative lors de toutes les visites de suivi. Les difficultés de compréhension étaient associées indépendamment au sexe féminin (rapport des cotes, RC : 0,29 ; intervalle de confiance de 95%, IC : 0,12–0,70) et au fait de résider dans la province de Jahaly plutôt que dans celle de Basse (RC : 0,33 ; IC de 95% : 0,13–0,82). Aucune association indépendante significative avec le niveau d'éducation n'a été observée. Le risque de voir le score de compréhension d'un participant baisser de moitié par rapport à sa valeur initiale était plus faible dans la branche d'intervention (ratio de risque 0,22, IC de 95% : 0,16-0,31). Globalement, 70% (42/60) des participants du groupe de discussion de la branche d'intervention ont trouvé l'outil multimédia clair et facile à comprendre.

**Conclusion:**

L'outil multimédia de consentement éclairé a sensiblement amélioré la compréhension et la mémorisation des informations sur le consentement par les participants à des recherches ayant un faible niveau d'alphabétisation.

## Resumen

**Objetivo:**

Evaluar la eficacia de una herramienta multimedia para el consentimiento informado para los adultos que participan en un ensayo clínico en Gambia.

**Métodos:**

Los adultos elegibles para ser incluidos en un ensayo de los tratamientos de la malaria (*n* = 311) fueron aleatorizados para recibir la información necesaria para el consentimiento informado mediante una herramienta multimedia (brazo de intervención) o un procedimiento estándar (brazo de control). Se utilizó un cuestionario de audio electrónico para evaluar la comprensión de los participantes del consentimiento informado. Esto se realizó inmediatamente después de la obtención del consentimiento (el día 0) y en las visitas de seguimiento posteriores (días 7, 14, 21 y 28). La aceptación y facilidad de uso de la herramienta multimedia se evaluó en grupos muestrales.

**Resultados:**

En el día 0, la puntuación de comprensión media en el brazo de intervención fue del 64%, en comparación con el 40% en el brazo de control (*P* = 0,042). La diferencia siguió siendo significativa en todas las visitas de seguimiento. La peor comprensión se relacionó independientemente con el sexo femenino (cociente de posibilidades, CP: 0,29; 95% intervalo de confianza, IC: 0,12–0,70) y con los residentes en Jahaly en lugar de la provincia de Basse (CP: 0,33 (95% IC: 0,13–0,82). No hubo ninguna asociación independiente significativa con el nivel educativo. El riesgo de que la puntuación de comprensión de un participante se redujese a la mitad de la puntuación inicial era menor en el brazo de intervención (cociente de riesgos instantáneos 0,22, 95% IC: 0,16–0,31). En términos generales, el 70% (42/60) de los participantes del grupo muestral del brazo de intervención consideraron que la herramienta multimedia era clara y fácil de entender.

**Conclusión:**

Una herramienta multimedia para el consentimiento informado mejoró considerablemente la comprensión y retención de la información consentida por los participantes en la investigación con bajos niveles de alfabetización.

## ملخص

**الغرض:**

تقييم فاعلية أداة موافقة مستنيرة متعددة الوسائط لمشاركة البالغين في تجربة سريرية في غامبيا.

**الطريقة:**

تم اختيار البالغين المؤهلين للمشاركة في تجربة علاج الملاريا ( *العدد * = 311) بشكل عشوائي لتلقي المعلومات اللازمة للموافقة المستنيرة باستخدام أداة متعددة الوسائط (ذراع التدخل) أو إجراء معياري (ذراع السيطرة). تم استخدام استبيان صوتي محوسب لتقييم فهم المشاركين للموافقة المستنيرة. تم إجراء ذلك على الفور بعد الحصول على الموافقة (في يوم 0) وفي زيارات متابعة لاحقة (في أيام 7 و14 و21 و28). تم تقييم مقبولية وسهولة استخدام الأداة متعددة الوسائط في مجموعات التركيز.

**النتائج:**

في يوم 0، سجل متوسط درجة الفهم في ذراع التدخل 64% مقارنة بدرجة 40% في ذراع السيطرة ( *الاحتمال * = 0.042). ظل الاختلاف كبيراً في جميع زيارات المتابعة. ارتبط الفهم الأقل بشكل مستقل بالإناث (نسبة الاحتمال: 0.29؛ بنسبة أرجحية مقدارها 95%: 0.12 إلى 0.70) والمقيمين في جاهالي أكثر من مقاطعة باسي (نسبة الاحتمال: 0.33؛ بنسبة أرجحية مقدارها 95%: 0.13 إلى 0.82). لم يكن هناك ارتباط مستقل كبير بالمستوى التعليمي. كان خطر انخفاض درجة الفهم لدى المشارك إلى نصف القيمة الأولية أقل في ذراع التدخل (نسبة الخطورة 0.22، بنسبة أرجحية مقدارها 95%: 0.16 إلى 0.31). بشكل إجمالي، وجد 70% من المشاركين (42/60) بمجموعة التركيز في ذراع التدخل أن الأداة متعددة الوسائط واضحة وسهلة الفهم.

**الاستنتاج:**

طوّرت أداة الموافقة المستنيرة متعددة الوسائط بشدة من فهم معلومات الموافقة والاحتفاظ بها من جانب المشاركين بالبحث ممن لديهم مستويات منخفضة من الإلمام بالقراءة والكتابة. ****

## 摘要

**目的:**

旨在针对冈比亚境内参与临床试验的成年人评价多媒体知情同意工具的有效性。

**方法:**

随机选出有资格参与疟疾治疗试验 (*n* = 311) 的成年人，让其通过多媒体工具（干预组）或标准程序（控制组）接收知情同意所需的信息。使用经过电脑处理的音频调查问卷有助于评价参与者对知情同意的理解。获得同意后会立即（第 0 天）并在随后的后续访问（第 7、14、21 和 28 天）中完成这一过程。多媒体工具的可接受性和易用性会在重点小组中进行评价。

**结果:**

第 0 天，干预组的理解评分中值为 64%，控制组为 40% (*P* = 0.042)。在所有后续访问中保持显著差异。理解不足与女性性别具有独立相关性（让步比，或：0.29；95% 置信区间，CI：0.12–0.70）并且其住在加哈利 (Jahaly) 而非巴塞 (Basse) 省（或：0.33；95% CI：0.13–0.82）。与教育水平并无显著的独立相关性。干预组中参与者的理解评分在可能会下降至初始值一半这方面的风险较低（风险比为 0.22，95% CI：0.16–0.31）。整体来说，干预组中 70% (42/60) 的重点小组参与者能够明确多媒体工具并且容易理解。

**结论:**

多媒体知情同意工具能大大提高具有较低文化水平的研究参与者对知情信息的理解和记忆。

## Резюме

**Цель:**

Оценить эффективность мультимедийного инструмента, предназначенного для получения информированного согласия взрослых на участие в клиническом исследовании в Гамбии.

**Методы:**

Взрослые, соответствующие критериям для включения в исследование по лечению малярии (n = 311), были случайным образом распределены в две группы. Одна группа получала информацию, необходимую для их информированного согласия, с помощью специального мультимедийного инструмента (экспериментальная группа), в другой группе использовалась стандартная процедура (группа сравнения). Для оценки понимания информированного согласия участниками исследования использовался компьютерный аудиоопросник. Оценка проводилась сразу же после получения согласия участника (день 0) и во время последующих контрольных визитов (дни 7, 14, 21 и 28). Приемлемость и простота использования мультимедийного инструмента оценивались в фокус-группах.

**Результаты:**

В день 0 средний балл понимания в экспериментальной группе составил 64 % по сравнению с 40 % в группе сравнения (P = 0,042). Разница оставалась значительной по результатам всех последующих визитов. Более низкий уровень понимания отмечался среди женщин (отношение шансов, ОШ: 0,29; 95% доверительный интервал, ДИ: 0,12–0,70) и проживающих в Джахалы в сравнении с провинцией Бассе (ОШ: 0,33; 95% ДИ: 0,13–0,82). Существенная зависимость от уровня образования не наблюдалась. Риск того, что уровень понимания участником сократится в два раза в сравнении с первоначальной оценкой, в экспериментальной группе был ниже (отношение рисков 0,22, 95% ДИ: 0,16–0,31). В целом 70 % (42/60) участников фокус-группы из экспериментальной группы заявили, что мультимедийный инструмент является понятным и простым в использовании.

**Вывод:**

Мультимедийный инструмент для получения информированного согласия значительно улучшил понимание и запоминание условий согласия среди участников с низким уровнем грамотности.

## Introduction

Clinical trial participants in sub-Saharan Africa often have limited understanding of the study information provided during the informed consent process. Low literacy and the difficulty of presenting information in local languages with no standard written form are contributory factors.^1^^,^^2^ Nevertheless, international ethics guidelines^3^^,^^4^ require informed consent to be obtained in a manner that can be understood by individuals volunteering for clinical studies. Moreover, the Declaration of Helsinki states that special attention should be given to the specific information needs of participants and to the methods used to deliver that information.^4^ Consequently, study information must be provided in a medium and language understood by potential participants. However, informed consent documents are usually written in an official national language, often a common international language. In countries such as the Gambia, where local languages have no standard written form, translating documents into the local language and back-translating into the national language (English, in this case), to check consistency is both impractical and inaccurate.^2^

Comprehension of consent information is essential for protecting study participants’ rights and for complying with the principles of good clinical practice. In sub-Saharan Africa, an increasing number of clinical trials are being conducted in populations that are vulnerable to exploitation because of poverty, illiteracy, social exclusion or poor access to health care.^5^^,^^6^ In particular, illiterate participants may not understand research concepts, which could undermine their ability to give truly informed consent.^5^ Comprehension could be improved using multimedia consent tools that have been effective for communicating crucial research information in developed countries.^7^^–^^9^ Moreover, empirical studies indicate that such tools provide an alternative means of presenting study information to vulnerable groups.^10^^,^^11^ The effectiveness of multimedia consent tools among clinical trial participants with low English-language literacy in Africa has not been determined.

Previously in the Gambia, we developed and validated both a multimedia tool for providing study information to clinical trial participants^12^ and a computerized, audio questionnaire for assessing their comprehension of informed consent.^13^ Here we report on the acceptability, ease of use and effectiveness of the multimedia tool among participants in a malaria treatment trial in the Gambia.

## Methods

We conducted a randomized controlled trial in Basse and Jahaly Provinces in the Upper River Region and Central River Region, respectively, of the Gambia from 15 August 2013 to 12 March 2014. The study was nested within a parent study – the PRINOGAM trial.^14^ The aim of this trial was to determine the optimal dosage of primaquine required to clear gametocytes and block disease transmission in asymptomatic malaria carriers. Participants in the PRINOGAM trial were aged 1 year or older and were seen on the day of inclusion (day 0, the baseline) and on days 3, 7, 14, 21, 28, 35 and 42. In the study areas, most residents were subsistence farmers and the adult literacy rate was about 50%.^15^

To be included in our study, individuals had to be eligible for the PRINOGAM trial, to be aged 18 years or older, to speak and understand one of the three major Gambian languages (i.e. Mandinka, Fula or Wolof) and to have no obvious communication, visual or cognitive impairments. Since a systematic review showed that, on average, 47% of participants in African studies understood basic research concepts,^1^ we estimated that a study with a 90% power to detect a 20% difference between intervention and control arms at the 5% significance level for a two-sided test would require 137 participants in each arm. On assuming a 10% attrition rate, we estimated that approximately 150 participants would be required in each group. In our study, participants were randomly assigned to the intervention or control arm on day 0 of the PRINOGAM trial, at the time of treatment randomization. An independent statistician used the RANDI2 web-based application (available at: http://dschrimpf.github.io/randi3/) to generate a randomization list for each trial site and participants were allocated to the intervention or control arm at a 1:1 ratio with a block size of four. In addition, participants were stratified by age group and sex.

### Intervention

The multimedia informed consent tool has been reported in detail elsewhere.^12^ Briefly, it contained information from the PRINOGAM consent document under the headings: (i) introduction; (ii) reason for the study; (iii) what glucose-6-phosphate dehydrogenase deficiency is; (iv) how to take part; (v) what happens if you take part; (vi) what blood tests are performed; (vii) what the side-effects and possible risks of taking part are; (viii) what the potential benefits are; (ix) how taking part is kept confidential; (x) who carried out an ethical review of the study; and (xi) who to contact if you have questions. The information in each section was presented in a context-specific visual form by members of the clinical trial team acting out various scenes after training. Video recordings were made by a multimedia expert and voice-overs were added separately in the three main Gambian languages. Adverse events of the study drugs that could not be adequately presented in the acted scenes, such as headache, diarrhoea or the passage of dark-coloured urine, were illustrated using animations.^12^ In addition, the multimedia tool was tailored to the cultural and linguistic diversity of the Gambian population. A digital versatile disc (DVD) incorporating the three language versions was produced and uploaded onto laptop computers. For the intervention arm of our study, a trained field assistant selected the language preferred by each participant and played the DVD using a laptop computer in a quiet room. Individuals consented to participating in the trial by either signing or thumb-printing the consent form in the presence of an impartial witness.

In the control arm, clinical trial information was presented using the current standard practice accepted by the national ethics committee in the Gambia (chair of the Gambia’s National Ethics Committee, personal communication, 12 October 2010). In the absence of acceptable written versions of the local languages, the study’s principal investigator trained field staff, who were native speakers of the major local languages, on the correct interpretation of the English version of the participants’ information sheet. Subsequently, the study information was presented verbally to prospective participants during discussions on informed consent. Again, consent was given by either signing or thumb-printing the consent form.^2^^,^^16^

### Assessment

We have previously shown that the computerized, audio, informed consent comprehension questionnaire is a reliable and valid tool for assessing comprehension of informed consent among Gambian trial participants.^13^ It comprises 26 questions in the three major Gambian languages: nine open-ended, seven closed-ended and 10 multiple-choice questions. The questionnaire was administered using laptop computers by trained interviewers who entered participants’ responses to each question. Responses were automatically recorded in the questionnaire computer database.

Comprehension was assessed on the basis of recall and understanding.^17^ Recall relates to the participant’s ability to correctly answer closed-ended and multiple-choice questions. Understanding is defined as the participant’s ability to correctly interpret or respond to open-ended questions. Our primary study outcome was comprehension of consent information as indicated by the participant’s questionnaire score, expressed as a percentage, on day 0 at study inclusion. Secondary outcomes were comprehension on days 7, 14, 21 and 28.

In addition, 119 randomly selected participants took part in focus group discussions on day 35, to further explore understanding of the PRINOGAM trial and to evaluate the acceptability and ease of use of the multimedia tool. Ten focus group discussion sessions were held in Basse Province; only six were held in Jahaly Province because there were fewer participants. Seven or eight participants were invited to each session and participants were segregated by sex so they could express their views more easily. The sessions were facilitated using a specially designed focus group discussion guide (available from corresponding author). Audio recordings of the sessions were transcribed into English by three translators and the accuracy of the translations was confirmed by independent translators fluent in the local languages and English. The transcribed texts were analysed using NVivo version 10.0 (QSR International Pty. Ltd, Doncaster, Australia) and the main themes that emerged were coded line by line to elucidate their meanings. The themes were subsequently sorted and collated into categories and subcategories and themes from the two sites were compared, integrated and refined. Finally, selected quotations from participants were used to illustrate differences in understanding of consent information between the intervention and control arms.

### Statistical analysis

Because data on comprehension of consent information were not normally distributed, we compared the median and interquartile range of participants’ comprehension scores at each visit in the two study arms. Associations between participants’ characteristics and baseline comprehension scores were assessed using the Mann–Whitney *U* test if the characteristic was classified using two categories and the Kruskal–Wallis test if more than two categories were used. We used forward stepwise variable selection in a multivariate logistic regression analysis to identify factors that were associated with comprehension on day 0. For this purpose, we reclassified the comprehension scores as a binary variable equal to 2 (below median) or 1 (median or above). Since participants were recruited at two different sites, we investigated the effect of clustering on comprehension using a mixed-effects model. Survival analysis was used to extrapolate the decline in comprehension scores beyond the end of the study follow-up. A *P*-value less than 0.05 was regarded as statistically significant. All statistical calculations were performed using Stata version 12.1 (StataCorp. LP, College Station, United States of America).

Approval was obtained from the ethics committee of the London School of Hygiene & Tropical Medicine in the United Kingdom of Great Britain and Northern Ireland and the Gambian Government–Medical Research Council Joint Ethics Committee. The trial was registered with the Pan African Clinical Trials Registry (PACTR 201402000775274).

## Results

**Fig. 1:**
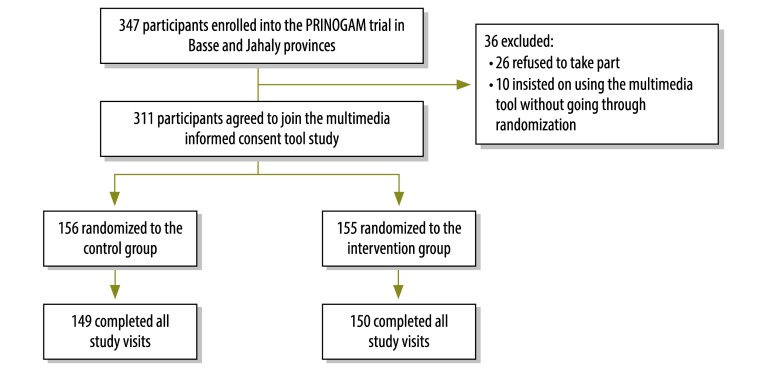
Flowchart for participants in the study of comprehension of informed consent, the Gambia, 2013–2014

Of the 347 participants enrolled in the PRINOGAM trial, 26 refused to take part (7.5%) in the study of the multimedia informed consent tool. Most of those who refused said they did not have time to wait because of domestic demands. In addition, 10 participants (2.9%) insisted on using the multimedia tool without going through randomization, most likely because they had heard about the tool through friends or family already enrolled in the study. After excluding these 36 participants, 311 were enrolled in the study and included in final analysis (Fig. 1). Table 1 (available at: http://www.who.int/bulletin/volumes/93/5/14-146159) shows there was no significant difference in demographic characteristics at baseline between participants in the two study arms. The playing time of the multimedia DVD was 19.4 minutes and the standard consent process took 30 to 35 minutes depending on the communication skills and experience of the research assistant providing the information. On average, question-and-answer sessions after the consent interviews took 32 minutes.

**Table 1:** Sociodemographic characteristics of study participants in randomized controlled trial of a multimedia consent tool, the Gambia, 2013–2014

Characteristic	No. (%)		*P*
	Intervention arm^a^ (*n* = 155)	Control arm^a^ (*n* = 156)	
**Age, years**			0.247
18–25	23 (14.8)	35 (22.4)	NA
26–33	50 (32.3)	44 (28.2)	NA
34–41	40 (25.8)	35 (22.4)	NA
42–49	28 (18.1)	34 (21.8)	NA
> 49	14 (9.0)	8 (5.1)	NA
**Sex**			0.692
Female	96 (61.9)	100 (64.1)	NA
Male	59 (38.1)	56 (35.9)	NA
**Place of residence**			0.443
Basse province	102 (65.8)	109 (69.9)	NA
Jahaly province	53 (34.2)	47 (30.1)	NA
**Ethnicity**			0.666
Mandinka	75 (48.4)	81 (51.9)	NA
Fula	66 (42.6)	62 (39.7)	NA
Wolof	8 (5.2)	5 (3.2)	NA
Sarahule	5 (3.2)	7 (4.5)	NA
Manjago	1 (0.7)	1 (0.6)	NA
**Education^b^**			0.097
Formal education	41 (26.5)	29 (18.6)	NA
No formal education	114 (73.5)	127 (81.4)	NA
**Religious affiliation**			0.995
Islam	153 (98.7)	154 (98.7)	NA
Christianity	2 (1.3)	2 (1.3)	NA
**Previous clinical trial participation**			0.071
Yes	14 (9.0)	28 (18.0)	NA
No	140 (90.3)	127 (81.4)	NA
Don’t know	1 (0.7)	1 (0.6)	NA

NA: not applicable.

^a^ In the intervention arm, study participants were informed about the clinical trial using a multimedia informed consent tool; in the control arm, information was provided using the current standard method for informed consent.

^b^ For the purposes of this study, the term “formal education” was defined as education based on an English-language curriculum that involved the completion of primary school, with or without 3 years of junior secondary school.

**Fig. 2:**
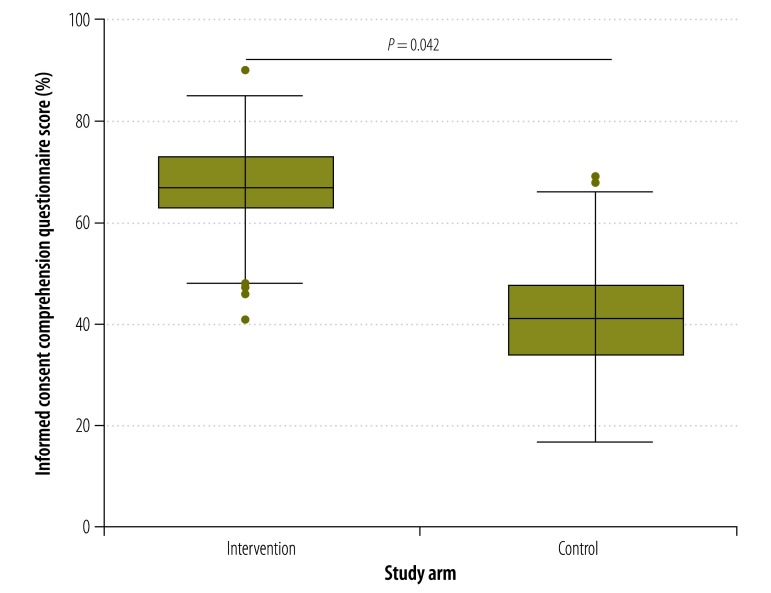
Informed consent comprehension questionnaire scores^a^ in intervention and control arms^b^ at baseline,^c^ the Gambia, 2013–2014

^a^ The box shows the values of the median and the first and third quartiles, the whiskers represent 1.5 times the interquartile range above and below the median and the small circles indicate outliers.

^b^ In the intervention arm, participants were informed about the trial using a multimedia informed consent tool; in the control arm, trial information was presented using current standard practice.

^c^ Baseline was day 0 of the parent treatment trial.

**Fig. 3:**
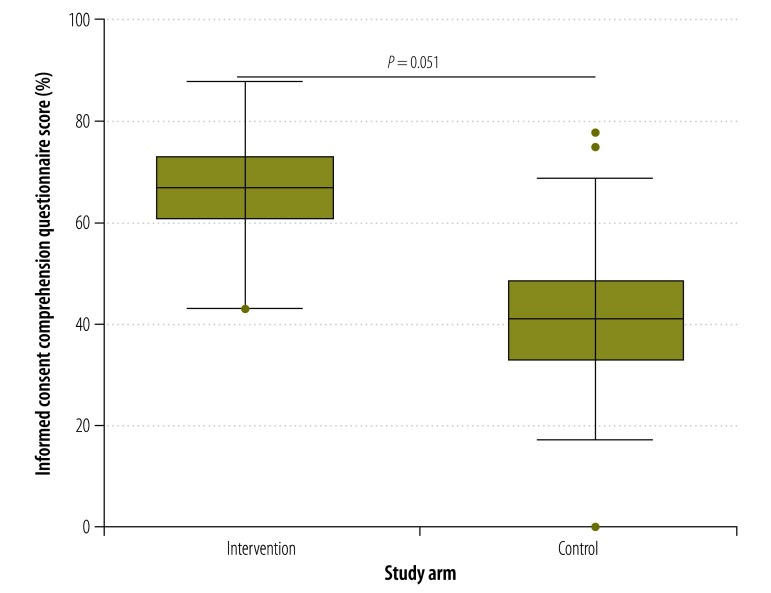
Informed consent comprehension questionnaire scores^a^ in intervention and control arms^b^ at day 7,^c^ the Gambia, 2013–2014

^a^ The box shows the values of the median and the first and third quartiles, the whiskers represent 1.5 times the interquartile range above and below the median and the small circles indicate outliers.

^b^ In the intervention arm, participants were informed about the trial using a multimedia informed consent tool; in the control arm, trial information was presented using current standard practice.

^c^ Day 7 of the parent treatment trial.

**Fig. 4:**
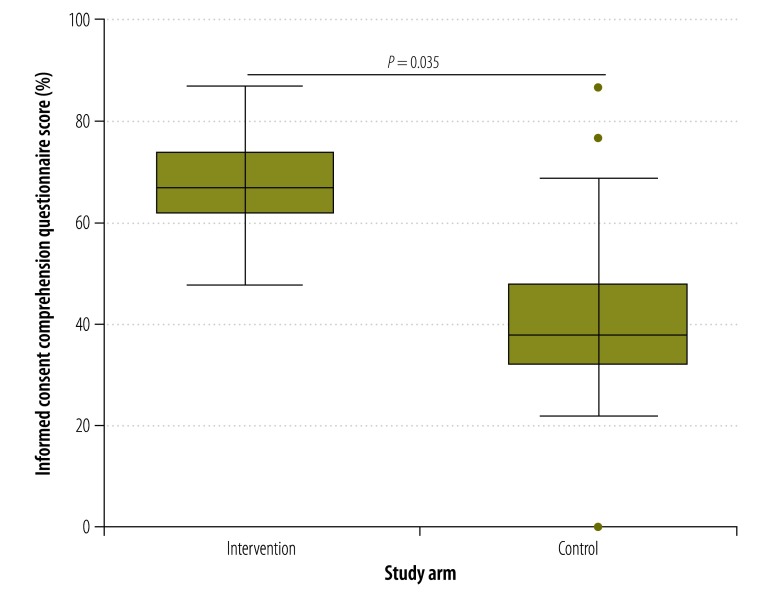
Informed consent comprehension questionnaire scores^a^ in intervention and control arms^b^ at day 14,^c^ the Gambia, 2013–2014

^a^ The box shows the values of the median and the first and third quartiles, the whiskers represent 1.5 times the interquartile range above and below the median and the small circles indicate outliers.

^b^ In the intervention arm, participants were informed about the trial using a multimedia informed consent tool; in the control arm, trial information was presented using current standard practice.

^c^ Day 14 of the parent treatment trial.

**Fig. 5:**
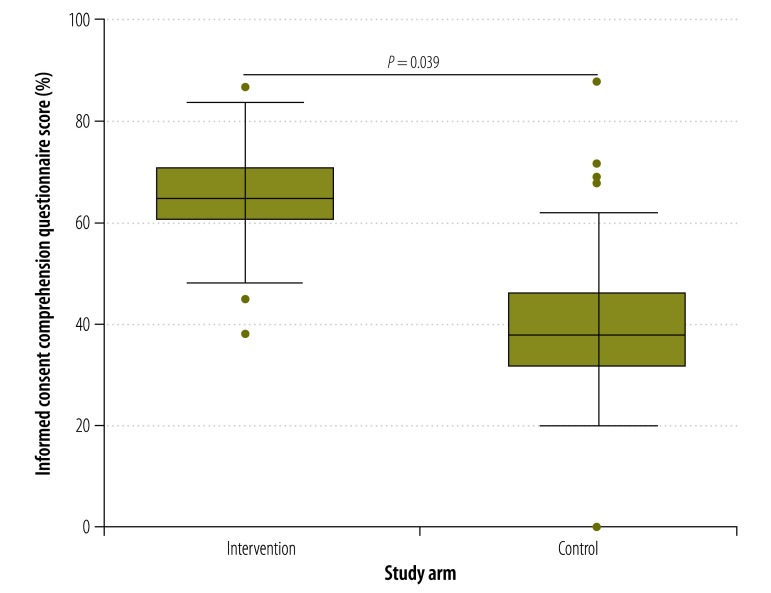
Informed consent comprehension questionnaire scores^a^ in intervention and control arms^b^ at day 21,^c^ the Gambia, 2013–2014

^a^ The box shows the values of the median and the first and third quartiles, the whiskers represent 1.5 times the interquartile range above and below the median and the small circles indicate outliers.

^b^ In the intervention arm, participants were informed about the trial using a multimedia informed consent tool; in the control arm, trial information was presented using current standard practice.

^c^ Day 21 of the parent treatment trial.

**Fig. 6:**
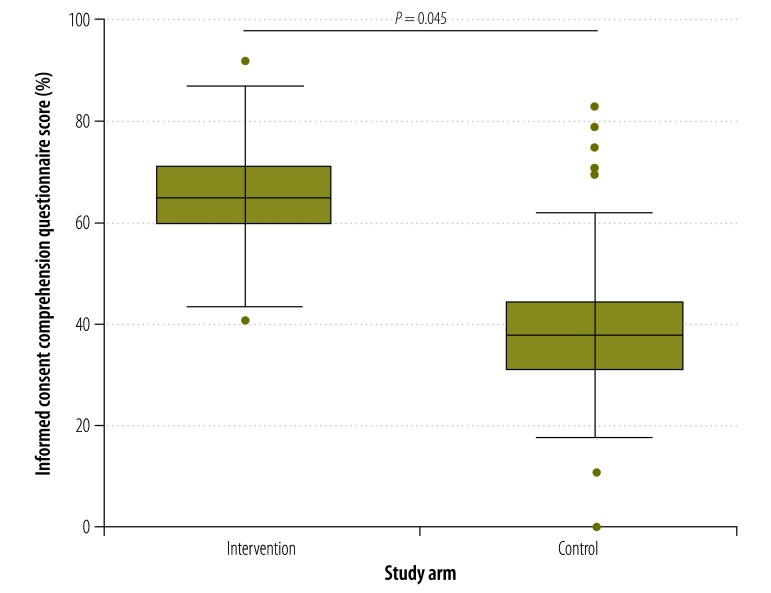
Informed consent comprehension questionnaire scores^a^ in intervention and control arms^b^ at day 28,^c^ the Gambia, 2013–2014

^a^ The box shows the values of the median and the first and third quartiles, the whiskers represent 1.5 times the interquartile range above and below the median and the small circles indicate outliers.

^b^ In the intervention arm, participants were informed about the trial using a multimedia informed consent tool; in the control arm, trial information was presented using current standard practice.

^c^ Day 28 of the parent treatment trial.

The median consent comprehension score of participants who used the multimedia tool was higher at all times points than those who received information using current standard practice. For example, at day 0 (Fig. 2), the median comprehension score in the intervention arm was 64% compared with 40% in the control arm (*P* = 0.042). The corresponding comparisons for days 7, 14, 21 and 28 are shown in Fig. 3, Fig. 4, Fig. 5 and Fig. 6, respectively. Table 2 shows that comprehension of informed consent at baseline in the two study arms combined was significantly greater in male participants, those who resided in Basse province and those who had received formal education based on an English-language curriculum. In addition, multivariate logistic regression analysis found that, after controlling for other covariates, poorer comprehension at baseline was significantly and independently associated with female sex (odds ratio, OR: 0.29; 95% confidence interval, CI: 0.12–0.70) and residing in Jahaly province (OR: 0.33; 95% CI: 0.13–0.82; Table 3). However, on applying the mixed-effects model, place of residence was no longer significantly associated with comprehension at baseline (OR: 0.85; 95% CI: 0.45–1.60; details available from authors). Survival analysis showed that the risk that a participant’s comprehension score would, during follow-up, drop to below 50% of that at day 0 was lower in the intervention arm than the control arm (hazard ratio 0.22, 95% CI: 0.16–0.31; details available from authors). Extrapolating beyond the end of follow-up indicated that the estimated median time for the comprehension score to drop below 50% was 67.0 days in the intervention arm compared with 40.6 days in the control arm (*P* < 0.0001). A summary of the economic and financial costs of developing and administering the multimedia informed consent tool is available from corresponding author.

**Table 2:** Influence of sociodemographic characteristics on comprehension of informed consent at baseline, the Gambia, 2013–2014

Characteristic	Informed consent comprehension questionnaire score, median (IQR)		*P^a^*
	Intervention (*n* = 155)	Control (*n* = 156)	
**Age, years**			0.54^b^
18–40	68.0 (63.0–73.0)	40.5 (33.5–46.5)	NA
≥ 41	65.0 (61.0–72.0)	43.0 (35.5–53.0)	NA
**Sex**			0.032^b^
Male	68.0 (65.0–73.0)	45.0 (38.0–51.0)	NA
Female	67.0 (61.0–72.0)	39.0 (33.0–46.0)	NA
**Place of residence**			0.021^b^
Basse province	67.5 (63.0–73.0)	44.0 (39.0–51.0)	NA
Jahaly province	67.0 (61.0–74.0)	33.0 (30.0–38.0)	NA
**Education^c^**			0.0049^b^
Formal education	66.5 (61.0–72.0)	40.0 (33.0–48.0)	NA
No formal education	70.0 (65.0–74.0)	45.0 (40.0–47.0)	NA
**Language of assessment**			0.92^d^
Mandinka	67.0 (64.0–73.0)	41.0 (33.0–47.0)	NA
Fula	69.0 (61.0–74.0)	38.0 (30.0–45.0)	NA
Wolof ^e^	67.5 (62.0–73.0)	42.0 (35.0–50.0)	NA
**Previous clinical trial participation**			0.21^d^
Yes	67.0 (63.0–73.0)	41.0 (34.0–48.0)	NA
No	69.0 (65.0–72.0)	40.0 (33.0–47.0)	NA
Don’t know	48.0 (48.0–48.0)	43.0 (43.0–43.0)	NA

IQR: interquartile range; NA: not applicable.

^a^ The *P*-value is for the significance of the influence of the sociodemographic characteristic on the participants’ comprehension of informed consent at baseline in the two arms combined.

^b^ The *P*-value for the significance of the association between the sociodemographic characteristic and baseline comprehension scores was assessed using the Mann–Whitney *U* test when there were two categories for the characteristic.

^c^ For the purposes of this study, the term “formal education” was defined as education based on an English-language curriculum that involved the completion of primary school, with or without 3 years of junior secondary school.

^d^ The *P*-value for the significance of the association between the sociodemographic characteristic and baseline comprehension scores was assessed using the Kruskal–Wallis test when there were more than two categories for the characteristic.

^e^ Included participants of Sarahule and Manjago ethnicities.

**Table 3:** Influence of sociodemographic characteristics on comprehension of informed consent at baseline, by multivariate logistic regression analysis, the Gambia, 2013–2014

Characteristic	Likelihood of better comprehension of informed consent,^a^ OR (95% CI)
Age group (18–40 years versus > 41 years)	1.41 (0.62–3.21)
Female versus male	0.29 (0.12–0.70)
Resident of Jahaly province vs Basse province	0.33 (0.13–0.82)
Formal education versus no formal education^b^	0.67 (0.23–1.93)
Assessment language: (Mandinka vs Wolof and Fula)	0.56 (0.29–1.08)
Previous trial participation versus no previous trial participation	1.07 (0.42–2.73)

CI: confidence interval; OR: odds ratio.

^a^ Better comprehension of informed consent was defined as an informed consent comprehension questionnaire score above the median for the intervention or control group, as appropriate.

^b^ For the purposes of this study, the term “formal education” was defined as education based on an English-language curriculum that involved the completion of primary school, with or without 3 years of junior secondary school.

### Focus group discussions

The 119 participants in the focus groups were aged between 23 and 47 years, 60 were in the intervention arm and 59 were in the control arm, 79 were female and 40 were male, and 56 resided in Basse Province and 63 resided in Jahaly Province. In Basse, 69.6% were female compared with 63.4% in Jahaly.

#### Informed consent

There was general consensus that signing or thumb-printing the consent form implied a commitment to participate in the research. One participant from Basse said, “When you put your hand in that paper, then you have promised to be part of the study.” However, there were divergent opinions on the right to withdraw. Whereas most participants in the control arm felt strongly that it was morally wrong to stop participating before the end of the study, the majority in the intervention arm stated that participants were free to leave at any time. One participant in the intervention arm said, “What we always think is that our doctors will be angry if we leave before the end of the study, but I now know after watching the ‘film’ that we have freedom to leave at any time, without telling them the reason for this… ”

Participants were unequivocal that incentives were needed to motivate them to join and stay in the trial. Whereas the majority considered the benefits of participation to be free medical care, a minority also wanted fertilizers and sponsorship of their children’s education. One said, “We appreciate all the good things you have done to care for us and our children, but the real help that we expect and will never be forgotten is to give us fertilizers for our crops and train our children to be like you....” Most participants in the control arm could not describe the risks involved, whereas participants in the intervention arm could often name the adverse events associated with the study medications, such as headache, abdominal pain, vomiting and diarrhoea. A male participant in the intervention arm described the passage of dark-coloured urine associated with haemolysis caused by primaquine in individuals with glucose-6-phosphate dehydrogenase deficiency, as urinating “*wonjo*”. “*Wonjo*” is a popular, local, dark-red coloured, hibiscus drink. Most participants in the intervention arm described the randomization procedure graphically and some participants in the control arm said randomization was done to ensure all participants had an equal chance of participating.

#### Multimedia informed consent tool

Overall, 70% (42/60) of focus group participants from the intervention arm thought the visual and verbal information presented through the DVD was clear and easy to understand. However, a few expressed reservations. One said, “Although I like the (computer) pictures and sounds, I prefer face-to-face talking. I can easily ask questions that are not clear to me and this will make me understand better.” Another said, “The Fula man (interpreter) on the computer (video) repeated the same information over and over, and this made everything boring to me.”

## Discussion

Our study’s findings confirm that use of a multimedia informed consent tool results in significantly better understanding of clinical trial information than the current standard method for obtaining consent. Participants using the multimedia tool achieved significantly higher consent comprehension scores at all study assessments. Moreover, results indicated that those who used the multimedia tool retained the study information significantly longer.

Although education, place of residence and sex were associated with participants’ comprehension scores at baseline, multivariate analysis found that the associations were significant only for place of residence and sex. This contrasts with findings of previous studies, which reported that educational level was an independent predictor of comprehension.^18^^,^^19^ The difference may have arisen because the large majority of our study participants had no formal education, which further strengthens the case for using multimedia tools to provide information to participants with low levels of literacy. Moreover, the multimedia tool was well received: in fact, during recruitment some participants insisted on being allocated to the intervention arm without undergoing formal randomization. Also, in focus group discussions, participants said they liked the way the study information was presented visually and verbally through the multimedia tool.

Our study adds to the emerging body of evidence that multimedia tools can increase trial participants’ comprehension of informed consent in Africa, particularly in areas where low literacy is common.^10^^,^^11^ In our study, research concepts that are known to be difficult to understand were clearly illustrated using video recordings and animations and clearly explained by sound tracks in three local languages. Furthermore, we nested our study within a malaria treatment trial to ensure that our findings were relevant to trials carried out in real-life settings, whereas previous studies conducted outside Africa adopted a simulated study design.^8^^,^^9^

The first limitation of our study was that, since our centre has been conducting research projects in the Gambia for more than 60 years, the local population was familiar with research projects. The effectiveness of the multimedia tool may be different in areas where the population is less familiar with research. Second, there was some clustering of participants: around two-thirds were recruited in Basse province, where the prevalence of asymptomatic malaria infection was higher than in Jahaly province. However, sociodemographic and epidemiological characteristics were similar at the two sites. Moreover, the mixed-effects model showed that place of residence had no significant effect on comprehension, which suggests that the effect of clustering was not significant.

In conclusion, our multimedia tool improved trial participants’ comprehension and retention of information about informed consent in an area of the Gambia with low levels of literacy. Such tools can help address the fundamental ethical challenge of obtaining informed consent from individuals in these settings.
